# Which Prognostic Index Is Most Appropriate in the Setting of Delayed Stereotactic Radiosurgery for Brain Metastases?

**DOI:** 10.3389/fonc.2016.00248

**Published:** 2016-11-21

**Authors:** Timothy Malouff, Nathan R. Bennion, Vivek Verma, Gabriel A. Martinez, Nathan Balkman, Abhijeet Bhirud, Tanner Smith, Chi Lin

**Affiliations:** ^1^School of Medicine, Creighton University, Omaha, NE, USA; ^2^Department of Radiation Oncology, University of Nebraska Medical Center, Omaha, NE, USA; ^3^Brigham Young University, Provo, UT, USA

**Keywords:** brain metastases, brain tumor, prognosis, radiation therapy, stereotactic radiosurgery

## Abstract

**Objectives:**

To determine if five commonly used prognostic indices (PIs) – recursive partitioning analysis (RPA), Score Index for Radiosurgery (SIR), Basic Score for Brain Metastases (BSBM), graded prognostic assessment (GPA), and the diagnosis-specific GPA – are valid following delay between diagnosis and treatment of brain metastases.

**Methods:**

In a single-institutional cohort, records of patients who underwent stereotactic radiosurgery (SRS) more than 30 days from diagnosis of brain metastases were collected, and five PI scores were calculated for each patient. For each PI, three score-based groupings were made to examine survival differences by means of adjusted log-rank analysis and area under the curve (AUC).

**Results:**

Of 121 patients with sufficient PI information, 72 underwent SRS more than 30 days after diagnosis. Median age and Karnofsky performance status were 60 years and 80, respectively. Forty-three (60%) patients had lung primaries. Prior to SRS, 38 (52.8%) and 12 (16.7%) patients underwent whole brain radiation therapy (WBRT) and surgery, respectively. Two (2.8%) patients underwent both WBRT and surgery prior to SRS. A median of two lesions were treated per SRS course. Median survival of the cohort was 9.0 months. Using adjusted log-rank analysis for pairwise comparison, BSBM and GPA showed significance between two out of the three prognostic groups, while the other scores showed either one or no significant differences on comparison. AUC demonstrated good applicability for BSBM, RPA, and GPA, although SIR was statistically less prognostic than the other PIs.

**Conclusion:**

The PIs analyzed in this study were applicable in the setting of delayed SRS. Although these data are hypothesis generating, they serve to encourage further analyses to validate a PI that is most optimal for these patients.

## Introduction

Accounting for over half of brain tumors, brain metastases are estimated to arise in 25–35% of all cancer patients (1, 2). Moreover, the incidence of metastatic brain disease has increased with more sensitive intracranial imaging and improved survival of patients with metastatic cancer as compared to the past (3–5). In the modern era, oncologic treatment for brain metastases includes whole brain radiation therapy (WBRT), surgical resection, stereotactic radiosurgery (SRS), systemic targeted therapy, or combinations of the aforementioned.

It is important, however, to appreciate those patients with brain metastases are undoubtedly part of a heterogeneous population, thus having direct implications on treatment paradigms. In order to better stratify patients based on expected prognosis, several numerical scoring systems have been proposed. These prognostic indices (PIs) may aid clinicians in selecting patients with longer expected survival who may potentially experience late effects of WBRT and hence benefit from SRS (6–10).

The Radiation Therapy Oncology Group (RTOG) recursive partitioning analysis (RPA) used data from three randomized trials (11–13) and was designed to explain differences in survival by dividing patients into prognostic subgroups (6). Several important variables were utilized in scoring, including but not limited to Karnofsky performance status (KPS), primary tumor control, age, and extracranial metastases. Next, the Score Index for Radiosurgery (SIR) was designed specifically for patients who undergo radiosurgery (7). In contrast to the RPA grouping, the SIR is a numerical scale applied to the sum of 5 prognostic factors, each having a category rated from 0 to 2. Although similar to the RPA (age, KPS, and extracranial disease), SIR also incorporates the number of metastases and the volume of the largest lesion. The Basic Score for Brain Metastases (BSBM) was derived by Lorenzoni et al. from a cohort of patients who underwent Gamma Knife radiosurgery (8). Though it largely does not account for tumor factors as in the SIR, it provides the most simplified analytic framework. This involves examining three categories (KPS, primary tumor control, and extracranial metastases) while using a numerical system in which total scores range from 0 to 3. Subsequently, the graded prognostic assessment (GPA) was a modification of the RPA based on data from the RTOG9508 trial, which demonstrated number of metastases to be prognostic (14, 15). In doing so, parameters such as systemic disease control (relatively subjective) and tumor volume (liable to change based on prior treatment) were removed. As a result, this PI includes age, KPS, number of intracranial metastases, and presence of extracranial metastases. Finally, owing to the heterogeneity of patients with similar GPA scores, a diagnosis-specific GPA (DSGPA) was formulated by examining individual tumor types as an independent prognostic factor (16). By subcategorizing patients by primary tumor, the DSGPA offered greater prognostic value for tumors such as melanoma, renal cell carcinoma, and gastrointestinal primaries.

Amidst the strong prognostic impact of these PIs, several shortcomings should be noted. Many of these PIs predate advances in screening and treatment for both intracranial and extracranial disease (17). Moreover, the recent rise of systemic and biologic therapy for metastatic cancer may affect measurement of various parameters and potentially alter accuracy of various PIs. Because the indices were validated to estimate survival specifically at the time of initial brain metastatic diagnosis, their prognostic value at certain time points after the initial diagnosis remains unclear. This is an important issue to address; the recent advent of several options to manage brain metastases often results in a delay in SRS as compared to prior studies. To date, there have been no publications examining PIs in patients undergoing delayed SRS. Therefore, in this study, we compared each of the five PIs (such as RPA, SIR, BSBM, GPA, and DSGPA) in an institutional cohort with brain metastases treated with SRS at least 30 days after being diagnosed with brain metastasis. In doing so, we aimed to examine which PIs were most appropriate for the growing number of patients who undergo delayed SRS (e.g., due to logistics, surgery, WBRT, systemic therapy, personal preferences, etc.).

## Materials and Methods

This study, approved by the Institutional Review Board, retrospectively reviewed all patients who completed a course of SRS between 2009 and 2014. Analyzed patients were those who had an interval of over 30 days between initial diagnosis of brain metastases and SRS treatment. This threshold was used in order to provide a “meaningfully delayed” time point which encompassed a sufficient sample size of patients. Data collected for each patient included specifics related to each particular patient, primary disease, metastatic brain disease, and treatment factors both prior to and after SRS. Patient factors included age, gender, symptoms and performance status. Disease factors included date of primary diagnosis, date diagnosed with brain metastases, primary site, histology, subtype if applicable, number of lesions, control of systemic disease, and presence of extracranial metastases. Treatment factors detailed use of corticosteroids, any previous treatments, and specifics of SRS delivery. Response to SRS was noted, as well as the date of last radiologic and clinical follow-up (including death).

Using these variables, RPA, SIR, BSBM, GPA, and DSGPA were calculated for each patient. For each PI, excluding DSGPA, patients were then organized into three groups based on score; numerical score cutoffs separating these groups was logically performed to ensure relatively uniform sample sizes in each group. Groupings were also based on similar expected survival based on prior publications, in efforts to decrease heterogeneity between grouped populations. For RPA scores, groups were made according to each numerical score (1, 2, and 3). Regarding SIR, scores of 1–3 were categorized as group 1, 4–6 were as group 2, and 7–9 as group 3. Similarly, a BSBM score of 0 was classified as group 1, a score of 1 corresponded with group 2, and scores of 2–3 were denoted as group 3. Regarding GPA, scores 0–1 were assigned to group 1, 1.5–2.5 to group 2, and 3–4 to group 3. DSGPA was calculated using the diagnostic information of the primary tumor from the following categories: non-small cell lung cancer (NSCLC), melanoma, breast, renal cell carcinoma, or gastrointestinal tumors. For the analysis of time from diagnosis to treatment as a prognostic factor, groupings were as follows: 30–44, 45–59, 60–89, and >90 days.

Kaplan–Meier survival curves for each group were then compiled for each PI. Survival was determined from the date of SRS to the date of last contact or death. Statistical analysis was performed using the SAS software, version 9.4 (SAS Institute Inc., Cary, NC, USA). Pairwise comparison of the groups was then preformed using the log-rank test and was adjusted for multiple comparisons. The Tarone–Ware test was used to account for non-proportionality. The area under the receiver operator characteristic curve was calculated for each index. Statistical significance was established using a *p*-value of <0.05.

## Results

Of the 121 patients who had sufficient information necessary for the calculation of the studied PIs (such as RPA, SIR, BSBM, GPA, and DSGPA), 72 patients had undergone SRS more than 30 days following initial diagnosis of brain metastases. Table 1 illustrates clinical and treatment characteristics of these patients. The median age and KPS at time of SRS were 60 (range 25–90) years and 80 (range 50–100), respectively. The median time from diagnosis to SRS was 2.9 (range 1–82) months. A median of 2 (range 1–5) tumors were treated per SRS course, at a median dose of 18 (range 13–24) Gy. The median interval between SRS and last follow-up, either by death or last clinical follow-up, was 6.7 (range 0.2–35.6) months. The median survival was 9.0 months, with 6- and 12-month survival rates of 64 and 31%, respectively. When all group 1s, 2s, and 3s for all PIs were analyzed together, unadjusted pairwise comparison showed significant differences in survival between groups 1 and 2 (*p* = 0.002), groups 1 and 3 (*p* < 0.001), and groups 2 and 3 (*p* = 0.045).

**Table 1:** **Clinical and treatment characteristics of the study population**.

Parameter	Value
Median (range) age, years	60 (25–90)
**Gender**	
Male	0.42 (58.3%)
Female	30 (41.7%)
**ECOG performance status**	
0	0.8 (11.1%)
1	39 (54.2%)
2	15 (20.8%)
3	9 (12.5%)
4	1 (1.4%)
**Karnofsky performance status**	
30	0.1 (1.4%)
40	0 (0.0%)
50	9 (12.5%)
60	5 (6.9%)
70	9 (12.5%)
80	20 (27.8%)
90	21 (29.2%)
100	7 (9.7%)
**Symptoms at presentation**	
Asymptomatic	0.12 (16.7%)
Headache	10 (13.9%)
Visual	9 (12.5%)
Sensorimotor	6 (8.3%)
Mental status	4 (5.6%)
Nausea	3 (4.2%)
Balance	3 (4.2%)
Seizure	2 (2.8%)
Other	6 (8.3%)
Unknown	17 (23.6%)
**Primary site**	
Lung	0.43 (59.7%)
Melanoma	9 (12.5%)
Breast	8 (11.1%)
Kidney	5 (6.9%)
Gastrointestinal	3 (4.2%)
Genitourinary	2 (2.8%)
Other	2 (2.8%)
**Receipt of surgery**	
For any other lesion(s)	0.19 (26.4%)
For lesion treated with SRS	12 (16.7%)
**Receipt of WBRT**	40 (55.6%)
30 Gy in 10 fractions	15 (20.8%)
37.5 Gy in 15 fractions	15 (20.8%)
Other	10 (25.0%)
**Previous SRS**	
For any other lesion(s)	0.4 (5.6%)
For lesion treated with SRS	0 (0.0%)
**Control of primary tumor**	
Yes	0.34 (47.2%)
No	38 (52.8%)
**Systemic disease status at treatment**	
Progressive	0.32 (44.4%)
Stable	29 (40.3%)
None	11 (15.3%)
**Presence of extracranial metastases**	
Yes	0.36 (50.0%)
No	36 (50.0%)
**Reason for delayed SRS**	
WBRT	0.38 (52.8%)
Surgery	12 (16.7%)
WBRT and surgery	2 (2.8%)
Targeted therapy	1 (1.4%)
Patient preference/other	19 (26.4%)
Median (range) lesions treated	2 (1–5)
Median (range) SRS dose (Gy)	18 (13–24)
Median (range) GTV volume (cc)	1.1 (0.03–15.2)
Median (range) PTV volume (cc)	1.9 (0.05–17.8)
Median (range) margin from GTV to PTV (mm)	1.0 (0.0–2.0)
**Immobilization technique**	
Frame-based	0.22 (30.6%)
Frameless	50 (69.4%)
**Response after SRS^a^**	
Complete response	0.11 (15.3%)
Partial response	9 (12.5%)
Stable disease	28 (38.9%)
Progressive disease	12 (16.7%)
Unknown	12 (16.7%)

*ECOG, Eastern Cooperative Oncology Group; SRS, stereotactic radiosurgery; WBRT, whole brain radiation therapy; GTV, gross tumor volume; PTV, planning target volume*.

*^a^Response after SRS was determined using the RECIST criteria for tumor response (18)*.

Regarding RPA, 15 patients were given a score of 1, 41 patients scored 2, and 16 patients scored 3. Based on the SIR index, 12 patients were assigned to group 1, 45 patients in group 2, and 15 patients in group 3. For BSBM, groups 1, 2, and 3 consisted of 9, 24, and 39 patients, respectively. In the analysis of GPA, group 1 consisted of 15 patients, group 2 had 42 patients, and group 3 included 15 patients.

**Figure 1:**
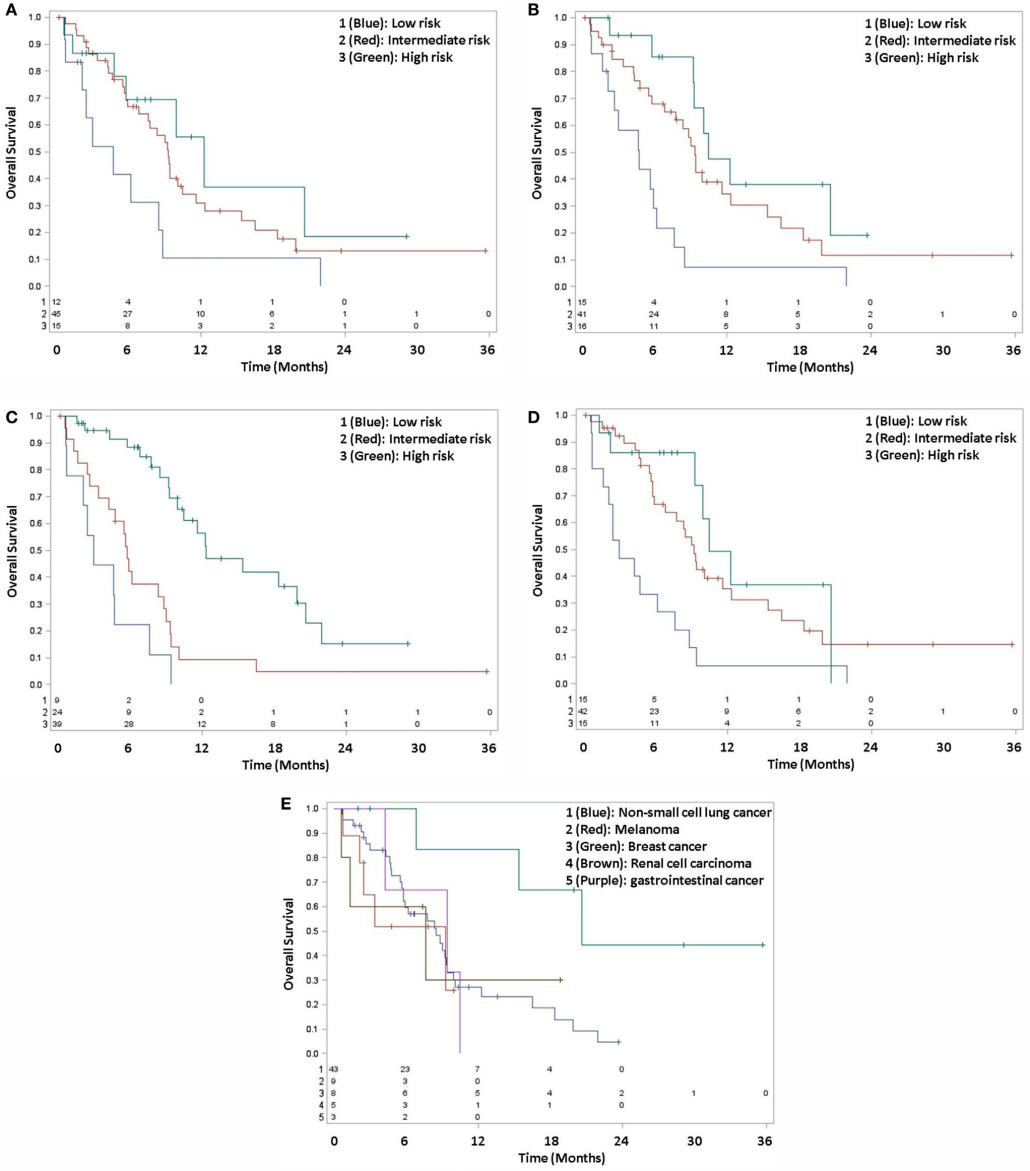
**Kaplan–Meier survival curves, grouped by SIR (A), RPA (B), BSBM (C), GPA (D), and DSGPA (E)**.

Adjusted pairwise comparison of the prognostic groupings in SIR found no statistical difference between any group (Figure 1A; Table 2). Although one comparison was statistically significant for RPA, the other two failed to be significant (Figure 1B). In contrast, two of the three comparisons were significant in BSBM and GPA, while the third was not significant (Figures 1C,D).

**Table 2:** **Survival and comparison between groups based on each prognostic index**.

Index	Median survival in months (95% CI)			*p*-Values of comparative survival		
	Group 1	Group 2	Group 3	Group 1 vs. 2	Group 1 vs. 3	Group 2 vs. 3
RPA	4.7 (1.6 to 6.2)	9.4 (6.8 to 12.4)	10.5 (9.2 to –)	0.13	<0.01	0.88
SIR	4.7 (0.7 to 8.5)	9.3 (6.8 to 10.5)	12.3 (4.8 to –)	0.38	0.09	0.99
BSBM	3.0 (0.7 to 7.6)	5.8 (3.4 to 8.7)	12.4 (9.3 to 20.6)	0.88	<0.01	<0.01
GPA	3.0 (0.7 to 6.2)	9.2 (5.9 to 12.4)	10.5 (9.3 to 20.6)	0.02	<0.01	1.00

*RPA, recursive partitioning analysis; SIR, Score Index for Radiosurgery; BSBM, Basic Score for Brain Metastases, GPA, graded prognostic assessment*.

For DSGPA, 43 patients were in group 1 (NSCLC), 9 patients were in group 2 (melanoma), 8 patients were in group 3 (breast cancer), 5 patients were in group 4 (renal cell carcinoma), and 3 patients were in group 5 (gastrointestinal). All comparisons by histologic type for DSGPA failed to reach statistical significance, as the adjusted log-rank *p*-values were >0.05 (Figure 1E).

**Figure 2:**
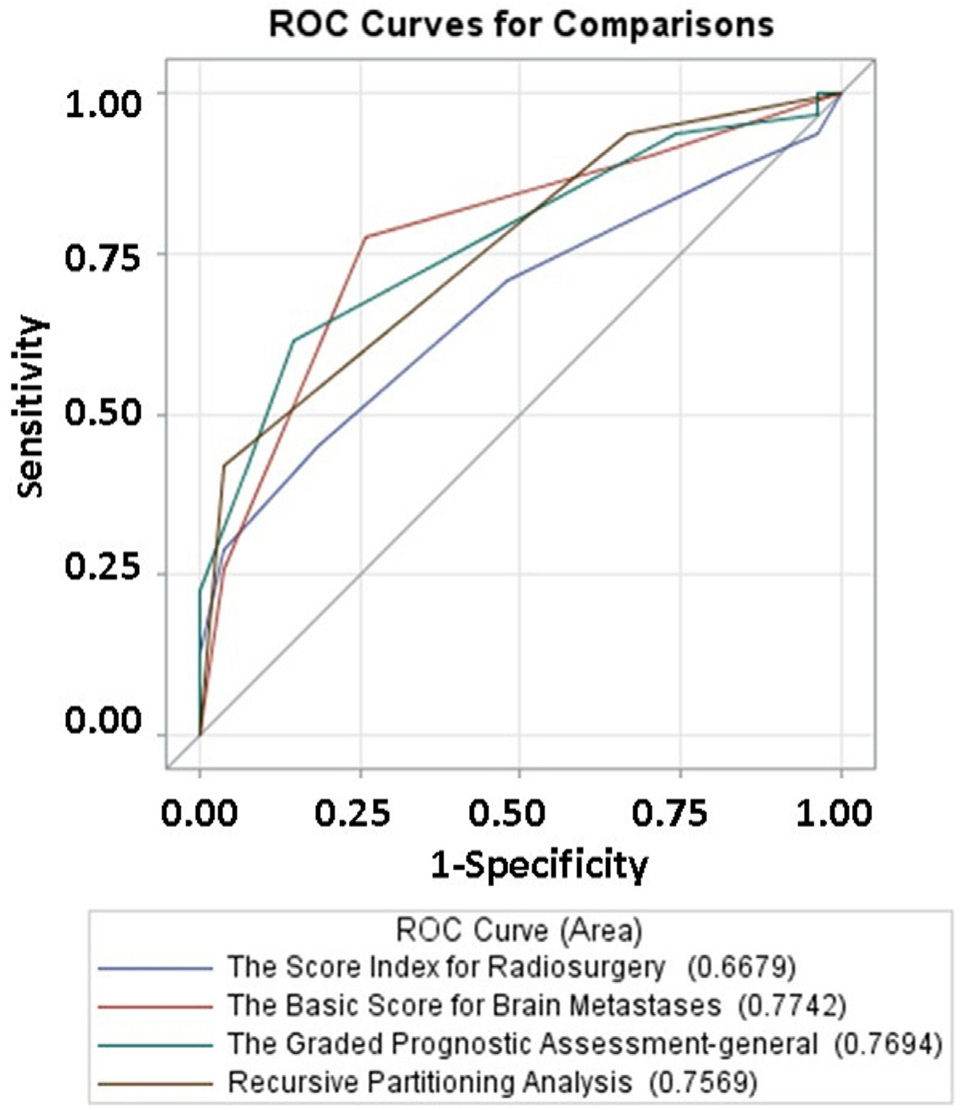
**Area under ROC curves for SIR, RPA, BSBM, and GPA**.

To better approximate prognostic values, the area under the curve (AUC) was calculated for SIR, BSBM, GPA, and RPA. Similar areas under the curve were observed with RPA (0.7569), BSBM (0.7742), and GPA (0.7694). However, SIR had a statistically significant decreased AUC (0.6679) as compared to GPA when (*p* = 0.04; Figure 2).

Several patient factors were analyzed to determine impact on prognosis. Forty patients received WBRT prior to SRS, while 32 patients did not receive previous WBRT. Those receiving WBRT had a worse median survival (6.16 months) compared to those who did not receive WBRT (10.10 months; *p* = 0.01). As expected, the 15 patients with a KPS < 70 at presentation had a worse median survival (4.66 months) than the 57 patients with a KPS of 70 or higher (9.41 months; *p* < 0.01). The number of brain metastases at presentation was found not to be prognostic, as the 22 patients with 1 brain metastasis (8.39 months) had similar median survivals to the 50 patients with more extensive disease (8.79 months; *p* = 0.58).

The impact of time between diagnosis and treatment as a prognostic factor was then analyzed. Thirteen patients received treatment between 30 and 44 days after diagnosis, with a median survival of 5.47 months. Eleven received treatment after 45–59 days, with a median survival of 5.77 months. Twelve patients received treatment after 60–89 days and had a median survival of 7.77 months, while 36 patients received treatment >90 days after diagnosis and had a median survival of 9.97 months. Although there was a trend toward longer survival with more time between diagnosis and treatment, there was no statistically significant difference between groups by the log-rank test (*p* = 0.20).

## Discussion

This is the first known study to examine the validity of several PIs (RPA, SIR, BSBM, GPA, and DSGPA) in an era of delayed SRS, with the increase in delays owing to more advances in treatment options prior to SRS. We demonstrate that all PIs show appropriate prognostic capabilities, with BSBM and GPA showing greater prognostic validity than RPA, SIR, or DSGPA.

Despite this, a main theme of our research is that certain PIs can be limited in the setting of delayed SRS. Similarly, Yamamoto et al. have detailed the limitations of RPA and BSBM as applying to patients undergoing repeat SRS (19). Additionally, a caveat associated with RPA is that it was originally designed for use in WBRT, while the other indices were developed for radiosurgery (20). Moreover, when using high-quality data from the RTOG database, Sperduto et al. demonstrated that median survivals often vastly differ from the original studies from which PIs were reported (14). For instance, the group observed median survival of 8.8 and 2.2 months in patients graded as SIR 8–10 and BSBM 3; median survivals of the original studies were 31.4 months and not reached, respectively (7, 8, 14). Furthermore, a recent study by Kondziolka and colleagues revealed that up to 45% of PI-based predictions differed by over 6 months (21). Furthermore, DSGPA can only be applied to the six subtypes of primary tumors, limiting its use for brain metastases arising from less common sites (19). Taken together, the specific patient population is integral to the applicability and prognostic impact of each PI.

Table 3 shows a comparison of our data with results from the original reports of each PI, recognizing that differing patient populations and grouping schemes make this comparison inherently faulty. In the worst prognostic group (group 1), GPA predicted survival most closely, and RPA the least. However, in group 2, GPA provided the greatest underestimation, while SIR was the numerically closest parameter. Group 3 has, as mentioned, been most liable to misestimations, but both GPA and BSBM provided close estimations in our patient population. Notably, other studies’ expected survivals underestimated those for both groups 1 and 2, likely a reflection of improved pre-SRS diagnosis and treatment. Collectively, this rough comparison further concludes that different PIs may be differentially accurate in various groups of patients, a notion that is not uncommon among other oncological prognostic tools.

**Table 3:** **Comparison of median survivals between this study and original studies for each parameter**.

Index	Median survival (months), group 1		Median survival (months), group 2		Median survival (months), group 3	
	Current study	Original study	Current study	Original study	Current study	Original study
RPA	4.7	2.3	9.4	4.2	10.5	7.1
SIR^a^	4.7	2.9	9.3	7.0	12.3	31.4
BSBM^b^	3.0	1.9	5.8	3.3	12.4	13.1+
GPA^c^	3.0	2.6	9.2	3.8	10.5	6.9–11.0

*RPA, recursive partitioning analysis; SIR, Score Index for Radiosurgery; BSBM, Basic Score for Brain Metastases, GPA, graded prognostic assessment*.

*^a^Original study grouped SIR by 1–3, 4–7, and 8–10 (current study with 1–3, 4–6, and 7–9)*.

*^b^Original study grouped BSBM by 0, 1, 2, and 3 (current study 0, 1, and 2–3)*.

*^c^Original study grouped GPA by 0–1, 1.5–2.5, 3, and 3.5–4 (current study 0–1, 1.5–2.5, and 3–4)*.

One of the major limitations of using PIs in any situation is the lack of consideration for genetic differences in tumors. For example, lapatinib has been found to affect survival of patients with brain metastases from HER2-positive breast cancers (22, 23). As such, one study developed and validated a nomogram for survival in breast cancer that factored in number of CNS metastases, size of largest brain metastasis, and biomarker status (24). Further research could adapt the nomograms to include a component for differences in survival from time of diagnosis to radiotherapeutic treatment, which could provide a more personalized prognostic timeline for patients considering a delay in receiving SRS.

While our study consisted of five of the most widely used prognostic scoring systems, there are other systems that may have value in the delayed setting. The Rotterdam score, which builds upon other PIs by including response to corticosteroids, systemic tumor activity, and serum lactate dehydrogenase, was validated in 1999 (25). However, the utilization of the Rotterdam score is less prevalent, owing to a dearth of consistent or sufficient data examining corticosteroid response (22). The Golden Grading System (GGS) was recently proposed using age, KPS score, and known extracranial metastases (26). Designed for SRS, surgery, and WBRT, the GGS does not take into consideration the number of brain metastases or the primary tumor site (20). In 2010, Rades et al. developed the RADES prognostic index, which factored in time of diagnosis from malignant disease to radiotherapy (27). Though this PI is potentially useful in the setting of delayed SRS, the index does not account for the difference between diagnosing brain metastases and diagnosing primary malignant disease, as brain metastases often occur months after the diagnosis of primary malignant disease. Furthermore, the interval between diagnosis and treatment of primary malignant disease was 6 months in the report, which is significantly longer than expected between diagnosis of brain metastases and SRS of brain metastases.

There are several limitations of this study worth mentioning. In addition to the retrospective nature of this work, its sample size may not result in optimal applicability. With only 72 patients in our study, our data may be underpowered to pick up differences between our prognostic groupings. Additionally, analysis of each individual prognostic score was underpowered, with only a few patients in each score. Although previous WBRT and poor performance status at presentation were found to have prognostic value, their analysis was limited due to small sample sizes in each subgroup. Furthermore, our definition of delayed was determined to be more than 30 days from diagnosis, based on the authors’ experiences. Being a single-institutional study, our study was underpowered to determine differences in more delayed settings (i.e., more than 60 days from diagnosis). Moreover, though all patients received SRS, which represents a more aggressive treatment course, it is important to note that we were unable to quantify the aggressiveness used in a systemic approach. Another potential area for bias is the grouping of our PI scores. Our grouping was largely based on sample size considerations, and though were similar to those used in the original publications, could result in non-trivial differences in accuracy. However, patterns of grouping are a bias present in any retrospective data, including the original publications. Direct comparison of DSGPA with other PIs was impractical, as the prognostic value of the DSGPA varies by disease site. For instance, a prognostic score of 3 in brain metastases from breast cancer is likely to have a different survival than a prognostic score of 3 in NSCLC. Finally, because PI scoring is designed to categorize a set of intrinsically heterogeneous patients, a very precise estimation of survival will continue to be an elusive target. The grouping of “patients undergoing delayed SRS” is no less heterogeneous, especially in light of various reasons for delaying SRS.

Future directions could examine endpoints for PIs in lieu of survival (22). While survival is an obvious and readily determinable endpoint, clinically relevant endpoints such as elsewhere brain failure and KPS decline may provide more relevant and precise information to patients. Furthermore, research could be performed to expand on the differences in survival among factors uniquely affecting patients with delayed SRS, which is becoming more common as newer therapies come to the forefront of oncologic management.

## Conclusion

As compared to the past, owing to improved diagnosis and treatment of brain metastases, delay between diagnosis and SRS is a relatively common scenario. PIs remain useful and accurate tools in the setting of delayed SRS, with some limitations. Therefore, careful selection of PIs is warranted. Though these results are hypothesis-generating, validation of PIs for delayed SRS using prospectively collected data is highly encouraged.

## Ethical Approval

The article does not contain any studies with human participants or animals performed by any of the authors.

## Informed Consent

Statement of informed consent was not applicable since the manuscript does not contain any patient data.

## Author Contributions

TM, NRB, and CL were involved in the conception of the work. TM, NRB, VV, GM, NB, AB, TS, and CL were involved in the acquisition, analysis, and interpretation of data and in drafting and revision of the manuscript. All the authors approve the final version and agree to be accountable for all aspects of the work.

## Conflict of Interest Statement

The authors declare that the research was conducted in the absence of any commercial or financial relationships that could be construed as a potential conflict of interest.
